# Nash Equilibrium of Social-Learning Agents in a Restless Multiarmed Bandit Game

**DOI:** 10.1038/s41598-017-01750-z

**Published:** 2017-05-16

**Authors:** Kazuaki Nakayama, Masato Hisakado, Shintaro Mori

**Affiliations:** 1 Department of Mathematics, Faculty of Science, Shinshu University, Asahi 3-1-1, Matsumoto, Nagano 390-8621 Japan; 2 Fintech Lab. LLC Meguro, Tokyo, 153-0051 Japan; 3 Department of Physics, Faculty of Science, Kitasato University, Kitasato 1-15-1, Sagamihara, Kanagawa 252-0373 Japan

## Abstract

We study a simple model for social-learning agents in a restless multiarmed bandit (rMAB). The bandit has one good arm that changes to a bad one with a certain probability. Each agent stochastically selects one of the two methods, random search (individual learning) or copying information from other agents (social learning), using which he/she seeks the good arm. Fitness of an agent is the probability to know the good arm in the steady state of the agent system. In this model, we explicitly construct the unique Nash equilibrium state and show that the corresponding strategy for each agent is an evolutionarily stable strategy (ESS) in the sense of Thomas. It is shown that the fitness of an agent with ESS is superior to that of an asocial learner when the success probability of social learning is greater than a threshold determined from the probability of success of individual learning, the probability of change of state of the rMAB, and the number of agents. The ESS Nash equilibrium is a solution to Rogers’ paradox.

## Introduction

One of the differences between human beings and other animals is that the former transfer their predecessors’ experience and wisdom in the form of knowledge^1^. Social learning—learning from the experience of others— is advantageous compared to individual learning^2–4^. Without social learning everybody would have to learn everything for themselves^2^. In other words, individual learning costs more than social learning does^2–4^. Therefore, Rogers’ finding that social learning is not necessarily more advantageous than individual learning is counterintuitive^5^. This is now called Rogers’ paradox.

Rogers’ conclusion seems very strange in light of our experience^4^. Several attempts have been made to solve Rogers’ paradox in social learning. Boyd and Richerson^2^ pointed out that Rogers’ paradox is *not* a paradox when the only benefit of social learning is to avoid learning costs. Further, on analysing two models where social learning reduces individual-learning costs and improves the information obtained through the latter, they concluded that social learning can be adaptive. Enquist *et al*.^3^ advocated a learning form called critical social learning, which is social learning supplemented by individual learning. They discussed using rate equations and succeeded in solving the paradox. Rendell *et al*.^4^ studied the relative merits of several learning strategies by using a spatially explicit stochastic model.

The concept of adaptive information filtering^3, 6^ has been proposed as key to the effective working of social learning. It indicates that each member effectively learns good-quality information provided by other members. For example, in a famous tournament by Rendell *et al*.^6^, discountmachine that did the most effective social learning won over the other strategies that combined individual learning and social learning.

In this study, we propose a stochastic model to solve Rogers’ paradox in the framework of a restless multiarmed bandit (rMAB) used in that tournament. The objective of this study is to analyse equilibrium social learning in an rMAB. An rMAB is analogous to the “one-armed bandit” slot machine but with multiple “arms”, each with a distinct payoff. We call an arm with a high payoff a good arm. The term “restless” means that the payoffs change randomly. Agents maximise their payoffs by exploiting an arm, searching for a good arm at random (individual learning), or copying an arm exploited by other agents (social learning). Because rMAB is simple in structure and its generality, we believe that it is an appropriate framework to consider Rogers’ paradox.

As a model for social-learning collectives, Bolton and Harris studied an agent system in a multi-armed bandit^7^. They assumed that the agents could know all information of other agents and obtained a socially optimal experiment (learning) strategy. In the present study, we consider the bounded rationality of agents, who can access the results of their respective choices only. In addition, we assume that the environment (i.e., the rMAB) changes randomly. We obtain the socially optimal and equilibrium learning strategies.

## Model

We make the model as simple as possible and incorporate the property of adaptive filtering of information into it. A mathematical overview of the model is given in the Methods section.

**Figure 1:**
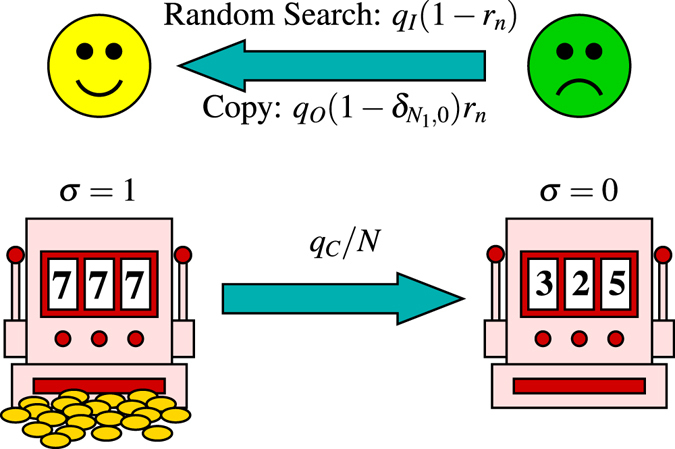
An agent and an rMAB. The number of agents who know a good arm is denoted as *N*_1_.

The rMAB has only one good arm and infinitely many bad arms. There are *N* agents labeled by *n* = 1, …, *N*. In each turn, an agent (say, agent *n*) is randomly chosen. He/she exploits his/her arm and obtains payoff 1 if he/she knows a good arm. If he/she does not know a good arm, he/she randomly searches for it (individual learning) with probability 1 − *r*_*n*_, or copies the information of other agents’ good arms (social learning) with probability *r*_*n*_. In the random search, the probability that he/she successfully finds a good arm is denoted as *q*_*I*_. On the other hand, we assume that the copy process succeeds with probability *q*_*O*_^8^ if there is at least one agent who knows a good arm, and fails if no agent knows a good arm. Then, with probability *q*_*C*_/*N*, the good arm changes to a bad one and another good arm appears. If a good arm changes to a bad one, the agents who knew the arm are forced to forget it and to know a bad one. See Fig. 1. The difference with our previous model^8^ is that there are *M* good arms in the previous model, whereas in the present model there is only one good arm.

Let *σ*_*n*_ be a random variable defined by

1 $${\sigma }_{n}=\{\begin{array}{ll}\mathrm{1,} & {\rm{if}}\,{\rm{agent}}\,n\,{\rm{knows}}\,{\rm{a}}\,{\rm{good}}\,{\rm{arm}},\\ \mathrm{0,} & {\rm{if}}\,{\rm{agent}}\,n\,{\rm{does}}\,{\rm{not}}\,{\rm{know}}\,{\rm{a}}\,{\rm{good}}\,{\rm{arm}}\mathrm{.}\end{array}$$

This is simply the payoff for agent *n*. For each turn *t*, we have a joint probability function *P*(*σ*_1_, …, *σ*_*N*_|*t*), which evolves in *t* according to the aforementioned rule. To exclude trivial results, we assume that *q*_*C*_, *q*_*I*_ and *q*_*O*_ are positive and that *r*_*n*_s are less than 1^8^. Then, in the long run, we have the unique steady probability function $P({\sigma }_{1},\cdots ,{\sigma }_{N})={\mathrm{lim}}_{t\to \infty }P({\sigma }_{1},\cdots ,{\sigma }_{N}|t)$. Now, we shall introduce the expected payoff for each agent in the steady state,

2 $${w}_{n}=E[{\sigma }_{n}]=\sum _{{\sigma }_{1}=\mathrm{0,1}}\cdots \sum _{{\sigma }_{N}\mathrm{=0,1}}P({\sigma }_{1},\cdots ,{\sigma }_{N}){\sigma }_{n},\,n=1,\cdots ,N\mathrm{.}$$

This quantity depends on parameters *N*, *q*_*C*_, *q*_*I*_, *q*_*O*_, and *r*_*n*_s. We regard *w*_*n*_ mainly as a function of *r*_*n*_s. We denote this function by *w*(*r*_*n*_, $\overline{r}$_*n*_), where

3 $$w(r,\overline{r})=\frac{1}{a+{q}_{I}+({q}_{O}-{q}_{I})r}\{{q}_{I}+({q}_{O}-{q}_{I})r-\frac{a{q}_{O}r}{a+(N-\mathrm{1)}{q}_{I}\mathrm{(1}-\overline{r})+{q}_{I}\mathrm{(1}-r)}\},$$

4 $$a=\frac{{q}_{C}}{1-{q}_{C}/N},$$

5 $${\overline{r}}_{n}=\frac{1}{N-1}\sum _{k\ne n}{r}_{k}=\frac{1}{N-1}\{\sum _{k\mathrm{=1}}^{N}{r}_{k}-{r}_{n}\}\mathrm{.}$$

Thus, we have *w*_*n*_ = *w*(*r*_*n*_, $\overline{r}$_*n*_) for each *n* = 1, …, *N*.

In this study, we treat *w*_*n*_ as the fitness for agent *n*.

## Results and Discussion

### Pure Strategies and Rogers’ Paradox

In the present study, the strategy of agent *n* refers to the social learning probability, *r*_*n*_. We call *r*_*n*_ = 0, 1 as pure strategies and 0 < *r*_*n*_ < 1 as mixed strategies.

First, we confirm that Rogers’ paradox occurs when agents adopt pure strategies. We shall divide *N* agents into two groups. The first group consists of *N*_*I*_ individual learners (*r*_*k*_ = 0, *k* = 1, …, *N*_*I*_). The second group consists of *N*_*S*_ = *N* − *N*_*I*_ social learners (*r*_*k*_ = 1, *k* = *N*_*I*_ + 1, …, *N*_*I*_ + *N*_*S*_). The corresponding fitness per agent, which we denote respectively as *w*_*I*_ and *w*_*S*_, are given by

6 $${w}_{I}=\frac{{q}_{I}}{a+{q}_{I}},\,{w}_{S}=\frac{{N}_{I}{q}_{I}{q}_{O}}{(a+{N}_{I}{q}_{I})(a+{q}_{O})},$$

where *a* is defined in equation (4).

When *q*_*O*_ ≤ *q*_*I*_, we have *w*_*I*_ > *w*_*S*_. Therefore, in this case, individual learning is always favourable over social learning.

**Figure 2:**
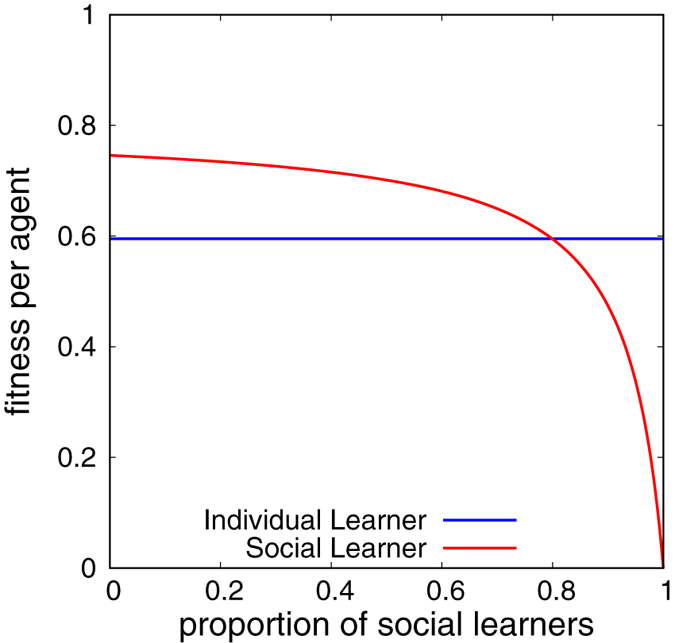
Plots of *w*_*I*_ and *w*_*S*_. Parameters: *N* = 10, *q*_*C*_ = 0.2, *q*_*I*_ = 0.3, *q*_*O*_ = 0.8.

Now, we consider the *q*_*O*_ > *q*_*I*_ case. Figure 2 is the plot of *w*_*I*_ and *w*_*S*_ for sufficiently large *N*.

When the proportion of social learners is small, social learning is effective. However, as the proportion of social learners increases, *w*_*S*_ monotonically decreases and tends to zero. Thus, Rogers’ paradox occurs.

It is important to note that *w*_*I*_ < *w*_*S*_ is true when *N*_*I*_/*N* is finite, with a sufficiently large *N*. This is because, as *N* → ∞, we have *w*_*I*_ → *q*_*I*_/(*q*_*C*_ + *q*_*I*_) and *w*_*S*_ → *q*_*O*_/(*q*_*C*_ + *q*_*O*_).

### Nash Equilibrium and Rogers’ Paradox

Let us assume that each agent adopts a mixed strategy, that is, for each *n* = 1, …, *N*, the social-learning probability, *r*_*n*_, is an arbitrary number between 0 and 1. This means that agent *n* performs social learning with probability *r*_*n*_ and individual learning with probability 1 − *r*_*n*_. The learning mode that he/she chooses would be decided stochastically and automatically.

We consider the *N*-tuple, (*r*_1_, …, *r*_*N*_), of the social-learning probabilities. This is a point in the *N*-dimensional unit cube *J* = [0, 1] × … × [0, 1]. *J* is regarded as the space of *N*-tuples of mixed strategies. For each point in *J*, a joint probability function *P*(*σ*_1_, …, *σ*_*N*_) is determined and an *N*-tuple, (*w*_1_, …, *w*_*N*_), of the fitness functions of the agents is calculated.

**Figure 3:**
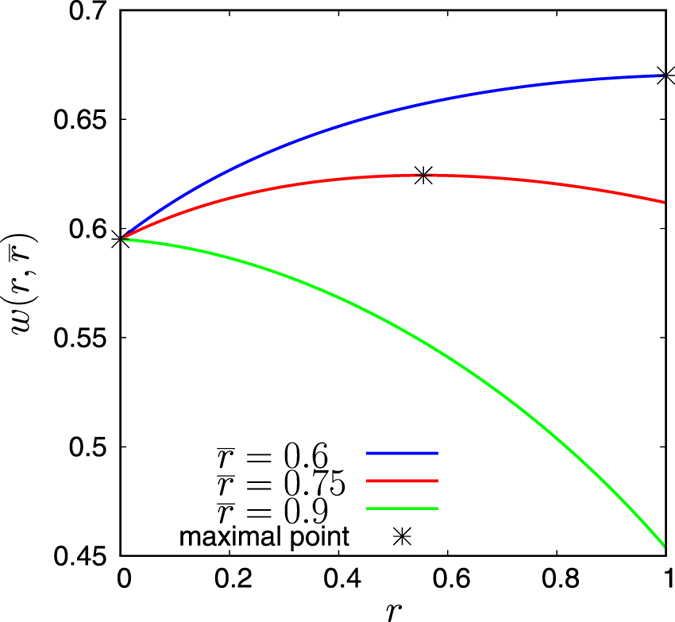
Plots of the function *w*(*r*, $\overline{r}$) versus *r* for various values of $\overline{r}$. Parameters: *N* = 10, *q*_*C*_ = 0.2, *q*_*I*_ = 0.3, *q*_*O*_ = 0.8.

Now, imagine that agent *n* maximises *w*_*n*_ by adjusting *r*_*n*_ for fixed *r*_*k*_s (*k* ≠ *n*). It is not difficult to show that the maximum point is unique (Fig. 3) and expressed as

7 $${r}_{n}=f({\overline{r}}_{n}),$$

where

8 $$f(r)=\{\begin{array}{cc}0 & {q}_{O}\le {q}_{I},\\ \min \,\mathrm{(1},\,\max \,\mathrm{(0,}\overline{f}(r))), & {q}_{O} > {q}_{I},\end{array}$$

9 $$\overline{f}(r)=\frac{-\zeta +\sqrt{{q}_{O}(N-\mathrm{1)(1}-r)}\sqrt{\zeta }}{{q}_{O}-{q}_{I}},$$

10 $$\zeta \equiv a+{q}_{I}+{q}_{I}(N-\mathrm{1)(1}-r\mathrm{).}$$

We note that *f*(*r*) → 0 as *q*_*O*_ → *q*_*I*_ + 0. Next, we introduce the function,

11 $$F({r}_{1},\cdots ,{r}_{N})=(f({\overline{r}}_{1}),\cdots ,f({\overline{r}}_{N}\mathrm{)).}$$

This is a continuous function mapping from the *N*-dimensional unit cube *J* into itself. As shown in the Methods section, the fixed point of *F* is unique and is on the diagonal line of *J*,

12 $${r}_{1}=\cdots ={r}_{N}={r}_{{\rm{Nash}}},$$

where *r*_Nash_ is a function of *q*_*C*_, *q*_*I*_, *q*_*O*_ and *N*. The value of *r*_Nash_ is explicitly given by

13 $${r}_{{\rm{Nash}}}=\{\begin{array}{cc}1-\eta , & ({q}_{O}-{q}_{I})N > a+{q}_{O},\\ \mathrm{0,} & ({q}_{O}-{q}_{I})N\le a+{q}_{O},\end{array}$$

where

14 $$\eta \equiv \frac{\mathrm{2(}a+{q}_{O}{)}^{2}}{({q}_{O}-{q}_{I}N)(a+{q}_{O})+(aN+{q}_{O})({q}_{O}-{q}_{I})+\sqrt{{D}_{1}}},$$

15 $$\begin{array}{c}{D}_{1}=(N-\mathrm{1)}{q}_{O}\{-\mathrm{(4}aN+3{q}_{O}N+{q}_{O}){q}_{I}^{2}\\ \,\,\,+\mathrm{2(3}a{q}_{O}N+2{q}_{O}^{2}N-2{a}^{2}-3a{q}_{O}){q}_{I}+a{q}_{O}(aN+3a+4{q}_{O})\}.\end{array}$$

**Figure 4:**
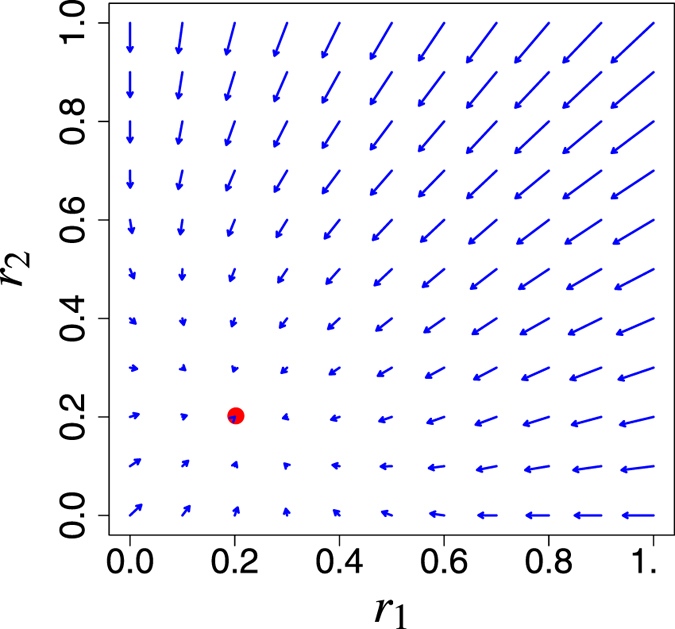
Plot of the vector field (*f*($\overline{r}$_1_) − *r*_1_, *f*($\overline{r}$_2_) − *r*_2_). The red spot is the Nash equilibrium point. Parameters: *q*_*C*_ = 0.1, *q*_*I*_ = 0.1, *q*_*O*_ = 0.8, and *N* = 2.

The entity *r*_Nash_ has the following properties (see the Methods section): (i) 0 ≤ *r*_Nash_ < 1, (ii) *r*_Nash_ → 0 as (*q*_*O*_ − *q*_*I*_)*N* − (*a* + *q*_*O*_) → 0, and (iii) the fixed point (*r*_Nash_, …, *r*_Nash_) is the unique Nash equilibrium point in *J*. Figure 4 is a schematic explanation of the Nash equilibrium point.

Moreover, the corresponding mixed strategy is an evolutionarily stable strategy (ESS)^9^ because the fixed point is a Nash equilibrium point in the strong sense,

16 $$w({r}_{{\rm{Nash}}},{r}_{{\rm{Nash}}}) > w(r,{r}_{{\rm{Nash}}}),\,{\rm{for}}\,{\rm{all}}\,{\rm{r}}\ne {{\rm{r}}}_{{\rm{Nash}}}\mathrm{.}$$

Further, it is an ESS in the sense of Thomas^10^, because the inequality,

17 $$w({r}_{{\rm{Nash}}},r) > w(r,r),\,{\rm{for}}\,{\rm{all}}\,{\rm{r}}\ne {{\rm{r}}}_{{\rm{Nash}}},$$

is true.

**Figure 5:**
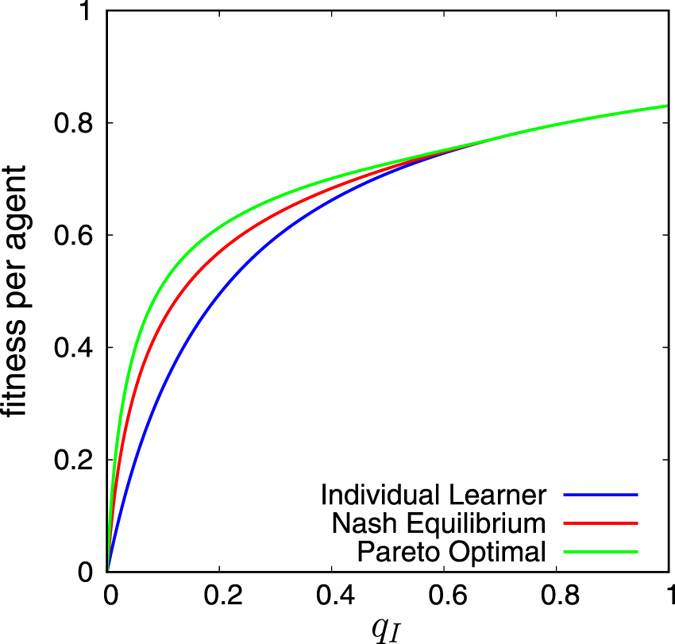
Plots of *w*_*I*_, *w*_*N*_, and *w*_*P*_ as functions of *q*_*I*_. Parameters: *N* = 10, *q*_*C*_ = 0.2, *q*_*O*_ = 0.8. Thus, $((N-\mathrm{1)}{q}_{O}-a)/N\simeq 0.7$.

Now, we consider the two fitness functions, *w*_*I*_ and *w*_*N*_ = *w* (*r*_Nash_, *r*_Nash_). As shown in the Methods section, the inequality *w*_*N*_ > *w*_*I*_ is correct if and only if (*q*_*O*_ − *q*_*I*_) *N* > *a* + *q*_*O*_. See also Fig. 5. The Nash equilibrium point is usually regarded as a stable point in the sense that no agent has an intention to change his/her strategy. Therefore, this inequality claims that the mixed strategy *r*_*n*_ = *r*_Nash_ (*n* = 1, …, *N*) can outperform the pure strategy of individual learning. This solves Rogers’ paradox. We note that the Nash equilibrium point is realised as a mixed strategy of social learning and individual learning.

### Pareto Optimality

Pareto optimality is an important concept alongside Nash equilibrium. Thus, we consider Pareto optimality in our model. We shall adopt a *natural* definition of the Pareto-optimal point in *J* as the maximum point of the function, ${\sum }_{k=1}^{n}{w}_{k}$. We can show that the maximum point is unique and is on the diagonal line of *J*,

18 $${r}_{1}=\cdots ={r}_{N}={r}_{{\rm{Pareto}}},$$

where *r*_Pareto_ is a function of *q*_*C*_, *q*_*I*_, *q*_*O*_, and *N*. The value of *r*_Pareto_ is explicitly given by

19 $${r}_{{\rm{Pareto}}}=\{\begin{array}{cc}\frac{(a+{q}_{I}N)X-(a+{q}_{I})Y}{{q}_{I}NX+({q}_{O}-{q}_{I})Y}, & ({q}_{O}-{q}_{I})N > a+{q}_{O},\\ \mathrm{0,} & ({q}_{O}-{q}_{I})N\le a+{q}_{O},\end{array}$$

where

20 $$X=\sqrt{(N-\mathrm{1)(}a+{q}_{O})({q}_{O}-{q}_{I})},$$

21 $$Y=\sqrt{(a+N{q}_{I})N{q}_{O}}\mathrm{.}$$

Further, *r*_Pareto_ has the following properties: (i) 0 ≤ *r*_Pareto_ < 1, (ii) *r*_Pareto_ → 0 as (*q*_*O*_ − *q*_*I*_)*N* − (*a* + *q*_*O*_) → 0, (iii) *r*_Pareto_ < *r*_Nash_ if and only if (*q*_*O*_ − *q*_*I*_)*N* > *a* + *q*_*O*_ (see the Methods section), and (iv) the point (*r*_Pareto_, …, *r*_Pareto_) is the Pareto-optimal point in *J*. Here, by Pareto optimality, we imply that the statement “if an agent succeeds to increase his/her fitness by changing his/her social-learning probability from *r*_Pareto_ to *r*_Pareto_ + *δr* by *δr* ≠ 0, then another agent’s fitness certainly decreases” is true. Such a *δr* exists when *r*_Pareto_ > 0 and no *δr* exists when *r*_Pareto_ = 0. The statement is correct in both cases.

We define the Pareto fitness function, *w*_*P*_ = *f*(*r*_Pareto_, *r*_Pareto_). Then, we have the inequality *w*_*P*_ > *w*_*N*_ if and only if (*q*_*O*_ − *q*_*I*_)*N* > *a* + *q*_*O*_ (see Fig. 5 and the Methods section). This is trivial by the definition of the Pareto-optimal point. Thus, we have established the relation among fitness functions,

22 $$\{\begin{array}{cc}{w}_{P} > {w}_{N} > {w}_{I}, & ({q}_{O}-{q}_{I})N > a+{q}_{O},\\ {w}_{P}={w}_{N}={w}_{I}, & ({q}_{O}-{q}_{I})N\le a+{q}_{O}\mathrm{.}\end{array}$$

## Concluding Remarks

We have proposed a stochastic model of *N* agents and an rMAB. The unique Nash equilibrium point in the mixed strategy space *J* has been presented and shown to be an ESS in the sense of Thomas^10^. The corresponding fitness *w*_*N*_ per agent is greater than the fitness *w*_*I*_ for an individual learner. This solves Rogers’ paradox.

In this study, we concentrated on steady states. This is valid if the system relaxes quickly to the steady state (see the Methods section). However, if *r*_*n*_s change faster than the relaxation to the steady state, it is an introduction of non-trivial dynamics. It may be possible that our system has a nice dynamics possessing the stable Nash equilibrium point.

As a future research subject, we propose an experimental study of human collectives in rMAB. There have been several attempts in this direction^11–13^, whose target has been the improvement of performance by social learning, that is, collective intelligence effect. Since we have shown that there is an ESS Nash equilibrium in the social-learning agents system in rMAB, it is interesting to experimentally examine whether the prediction is realised. As a first step, the interactive rMAB game might be a suitable environment where one human competes with many other mixed-strategy agents and *r* = *r*_Nash_. We can check whether the social-learning rate of people is the same with *r*_Nash_. Second, when many people compete, the Nash equilibrium emerges as the model parameter *q*_*I*_ changes. Meanwhile, we might be able to detect some phase-transitive behaviour^8^.

As for theoretical research, the stage of our analysis is far from mature. In the present work, we have studied the game of rMAB in the steady state of the system. However, when the relaxation time of the system discussed in the Methods section is not small enough, the assumption of steadiness is unrealistic in the laboratory experiment. Thus, we need to develop a *t*-dependent theory. It might be a difficult problem. We believe that the research direction is fruitful.

## Methods

### Mathematical Overview of the Model

For simplicity we use the following notation,

23 $$\vec{\sigma }=({\sigma }_{1},\cdots ,{\sigma }_{N}),\,\vec{0}=\mathrm{(0},\cdots ,\mathrm{0)},\,{\vec{e}}_{n}=\mathrm{(0},\cdots ,0,\mathop{\breve{1}}\limits^{n},0,\cdots ,\mathrm{0)},\,{\delta }_{{\vec{\sigma }}^{\text{'}},\vec{\sigma }}=\prod _{n\mathrm{=1}}^{N}{\delta }_{{\sigma }_{n}^{\text{'}},{\sigma }_{n}}\mathrm{.}$$

Our model develops in *t* according to an agent action and the subsequent state change of the rMAB. This is a Markov process^14^. The probability of change $\mathop{\sigma}\limits^{\longrightarrow}\to \vec{\sigma}^{\prime}$ is described by the transition probability matrix^14^,

24 $$T(\vec{\sigma }^{\prime} |\vec{\sigma })=(1-\frac{{q}_{C}}{N})\{(1-\sum _{n\mathrm{=1}}^{N}{p}_{n}(\vec{\sigma })){\delta }_{{\vec{\sigma }}^{\text{'}},\vec{\sigma }}+\sum _{n\mathrm{=1}}^{N}{p}_{n}(\vec{\sigma }){\delta }_{{\vec{\sigma }}^{\text{'}},\vec{\sigma }+{\vec{e}}_{n}}\}+\frac{{q}_{C}}{N}{\delta }_{{\vec{\sigma }}^{\text{'}},\vec{0}},$$

where

25 $${p}_{n}(\vec{\sigma })=\frac{{\delta }_{{\sigma }_{n}\mathrm{,0}}}{N}\{{r}_{n}\mathrm{(1}-{\delta }_{{N}_{1}\mathrm{,0}}){q}_{O}+\mathrm{(1}-{r}_{n}){q}_{I}\},\,{N}_{1}=\sum _{n\mathrm{=1}}^{N}{\sigma }_{n}\mathrm{.}$$

The joint probability function $P(\mathop{\sigma }\limits^{\longrightarrow}|t)=P({\sigma }_{1},\ldots ,{\sigma }_{N}|t)$ satisfies the Chapman-Kolmogorov equation^14^,

26 $$P(\vec{\sigma }|t+1)=\sum _{{\vec{\sigma }}^{\text{'}}}T(\vec{\sigma }|\vec{\sigma }^{\prime} )P(\vec{\sigma }^{\prime} |t)\mathrm{.}$$

Our assumption is that *q*_*C*_, *q*_*I*_, *q*_*O*_ > 0 and *r*_*n*_ < 1 (*n* = 1, …, *N*). In this case, the matrix *T* is shown to be irreducible and primitive^15^. Then, the Perron-Frobenius theory^15^ ensures that (i) *λ*_1_ = 1 is an eigenvalue of *T* of multiplicity 1 and the steady probability function *P*($\vec{\sigma }$) is a corresponding eigenvector, (ii) the set {|*λ*_*i*_|}_*i*≥2_ of absolute values of eigenvalues of *T* other than *λ*_1_ has an upper bound *ρ* < 1. When *r*_*n*_s are fixed, we have the time-homogeneous Markov process^14^, that is, the matrix *T* does not depend on *t*. Therefore, for any initial probability function $P(\vec{\sigma }|0)$, we have the unique limit $P(\vec{\sigma })={\mathrm{lim}}_{t \rightarrow \infty }P(\vec{\sigma }|t)$. Then, it is not difficult to derive equation (3) using $P(\vec{\sigma })$.

The convergence $P(\vec{\sigma }|t) \rightarrow P(\vec{\sigma })$ is exponential, $|P(\vec{\sigma }|t)-P(\vec{\sigma })|\sim {\rho }^{t}$. This means that the relaxation time is $\tau =-\,1/\,\mathrm{log}\,{\rho }^{-1}$. Thus, when no agent changes his/her social learning probability over a much longer period than *τ*, the fitness per agent per turn is almost exactly equal to the value of the function *w* in equation (3).

### Existence of a Fixed Point of *F*

Since the *N*-dimensional cube *J* = [0, 1] × … × [0, 1] is a compact, convex set and *F* is a continuous function mapping from *J* into itself, Brouwer’s fixed-point theorem^16^ guarantees that there exists a fixed point of *F* in *J*.

### A Fixed Point of *F* is a Nash Equilibrium Point, and Vice Versa

Let (*r*_1_, …, *r*_*N*_) be a fixed point of *F*, that is, *r*_*n*_ = *f*($\overline{r}$_*n*_) for each *n* = 1, …, *N*. Since *r* = *f*($\overline{r}$_*n*_) is the unique maximal point of *w*(*r*, $\overline{r}$_*n*_), we have *w*(*r*_*n*_ + *δr*, $\overline{r}$_*n*_) < *w*(*r*_*n*_, $\overline{r}$_*n*_) for each *n* = 1, …, *N* when *δr* ≠ 0. Thus, (*r*_1_, …, *r*_*N*_) is a Nash equilibrium point. Conversely, let (*r*_1_, …, *r*_*N*_) be a Nash equilibrium point, that is, *r* = *r*_*n*_ is a maximal point of *w*(*r*, $\overline{r}$_*n*_) for each *n* = 1, …, *N*. Since *r* = *f*($\overline{r}$_*n*_) is the unique maximal point of *w*(*r*, $\overline{r}$_*n*_) (see Fig. 3), we have *r*_*n*_ = *f*($\overline{r}$_*n*_). Thus, (*r*_1_, …, *r*_*N*_) is a fixed point of *F*.

### Uniqueness of the Fixed Point of *F*

When *q*_*O*_ ≤ *q*_*I*_, we have the unique fixed point (0, …, 0).

Next, we consider the *q*_*O*_ > *q*_*I*_ case.

Let (*r*_1_, …, *r*_*N*_) be a fixed point of *F*. Since $\overline{r}$_*n*_ = (*s* − *r*_*n*_)/(*N* − 1), $s={\sum }_{k=1}^{N}{r}_{k}$, all the *r*_*n*_s satisfy the common relation,

27 $$r=g(r)\equiv f(\frac{s-r}{N-1})\mathrm{.}$$

**Figure 6:**
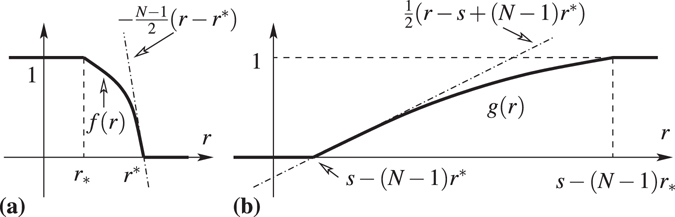
(**a**) Plot of the function *f*(*r*). (**b**) Plot of the function *g*(*r*).

Figure 6(b) is a plot of the function *g*(*r*).

This is a strictly increasing concave function for *s* − (*N* − 1)*r* ^*^ ≤ *r* ≤ *s* − (*N* − 1)r_*_, where

28 $${r}^{\ast }=1-\frac{a+{q}_{I}}{(N-\mathrm{1)(}{q}_{O}-{q}_{I})},$$

29 $${r}_{\ast }=1-\frac{-({q}_{O}(a-{q}_{I})-2a{q}_{I})+\sqrt{{D}_{2}}}{\mathrm{2(}N-\mathrm{1)}{q}_{I}({q}_{O}-{q}_{I})},$$

30 $${D}_{2}={({q}_{O}(a-{q}_{I})-2a{q}_{I})}^{2}+4{q}_{I}({q}_{O}-{q}_{I})(a+{q}_{O}{)}^{2}\mathrm{.}$$

It is not difficult to show that r_*_ < *r* ^*^ < 1. The maximum value of the derivative *g*′(*r*) is 1/2, which is realised at *r* = *s* − (*N* − 1)*r* ^*^. Thus, $\tilde{g}(r)=r-g(r)$ is a strictly increasing function such that $\tilde{g}\mathrm{(0)}\le 0\le \tilde{g}\mathrm{(1)}$. Therefore, there is only one zero, *r*_0_, of the function $\tilde{g}(r)$ in the interval 0 ≤ *r* ≤ 1. Then, we conclude that *r*_1_ = … = *r*_*N*_ = *r*_0_.

Now we have *s* = *Nr*_0_. Therefore, *r*_0_ is a solution of the equation,

31 $$r=f(r\mathrm{).}$$

Figure 6(a) is a plot of the function *f*(*r*). The function *f*(*r*) is a decreasing function. Thus, *h*(*r*) = *r* − *f*(*r*) is a strictly increasing function such that *h*(0) ≤ 0 ≤ *h*(1). Therefore, the function *h*(*r*) possesses only one zero, *r*_Nash_, such that 0 ≤ *r*_Nash_ < 1. Thus, we have *r*_0_ = *r*_Nash_. This proves the uniqueness of the Nash equilibrium point.

### Inequality *w*_*P*_ > *w*_*N*_ > *w*_*I*_

It is sufficient to consider the (*q*_*O*_ − *q*_*I*_)*N* > *a* + *q*_*O*_ case. Then, *r*_Nash_ satisfies $r=\overline{f}(r)$. We introduce the following function,

32 $$k(u)=(a+{q}_{O}){u}^{2}-\{({q}_{O}-{q}_{I}N)(a+{q}_{O})+(aN+{q}_{O})({q}_{O}-{q}_{I})\}u+({q}_{O}-{q}_{I})({q}_{O}-{q}_{I}{N}^{2}).$$

It is not difficult to check that 1/(1 − *r*_Nash_) is the larger root of *k*(*u*). We note that *k*(1) < 0.

Next, we define *r*_*I*_ as

33 $${r}_{I}=1-\frac{a+{q}_{O}}{({q}_{O}-{q}_{I})N}\mathrm{.}$$

This entity has the following properties: (i) 0 < *r*_*I*_ < 1, (ii) *w*(*r*_*I*_, *r*_*I*_) = *w*_*I*_, and (iii) *k* (1/(1 − *r*_*I*_)) > 0. On the other hand, it is elementary to show that *k* (1/(1 − *r*_Pareto_)) < 0. Thus, we conclude that *r*_Pareto_ < *r*_Nash_ < *r*_*I*_.

Now, *r*_Pareto_ is the maximal point of *w*(*r*, *r*). Therefore, we have the inequality *w*(*r*_Pareto_, *r*_Pareto_) > *w*(*r*_Nash_, *r*_Nash_) > *w*(*r*_*I*_, *r*_*I*_), that is, *w*_*P*_ > *w*_*N*_ > *w*_*I*_.
